# Biomechanical evaluation of a new femoral stem design for total hip replacement in a canine model

**DOI:** 10.1590/ACB360506

**Published:** 2021-06-21

**Authors:** Luís Guilherme de Faria, Bruno Watanabe Minto, Antonio Carlos Shimano, Ana Paula Macedo, Lucia Maria Izique Diogo, Rafael Manzini Dreibi, Matheus Nobile, Wanderley Severo Santos, Fernando Yoiti Kitamura Kawamoto, Guilherme Galhardo Franco, Luis Gustavo Gosuen Gonçalves Dias

**Affiliations:** 1 Fellow PhD degree. Postgraduate Program in Veterinary - Universidade Estadual Paulista “Julio de Mesquita Filho” - Jaboticabal (SP), Brazil.; 2 DVM, MSc, PhD. Department of Medicine and Locomotive Apparatus Rehabilitation - Medical School of Ribeirão Preto – Universidade de São Paulo, Ribeirao Preto (SP), Brazil.; 3 DVM, MSc. Department of Veterinary Clinic and Surgery - Faculty of Agrarian and Veterinary Sciences – Universidade Estadual Paulista “Julio de Mesquita Filho” - Jaboticabal (SP), Brazil.; 4 DVM. Department of Veterinary Clinic and Surgery - Faculty of Agrarian and Veterinary Sciences – Universidade Estadual Paulista “Julio de Mesquita Filho” - Jaboticabal (SP), Brazil.; 5 DVM. Department of Veterinary Clinic and Surgery – Universidade Federal Rural do Rio de Janeiro – Rio de Janeiro (RJ), Brazil.

**Keywords:** Hip Prosthesis, Dogs

## Abstract

**Purpose:**

To evaluate the biomechanical properties of a novel total hip replacement femoral stem.

**Methods:**

Eight pairs of femurs from dog cadavers were used. The femurs were separated into different groups. A novel femoral stem with a convex proximal portion (Stem B) was biomechanically evaluated and compared to awell-known veterinary collared stem (Stem A). Femoral stems were inserted into the contralateral femurs from the same dog, forming 16 constructs. A flexo-compression load was applied on the axial axis of each sample. Maximum strength, deflection, stiffness, and energy absorption were analysed.

**Results:**

Group B constructs showed significantly higher values (p ? 0.05) for the variables, except stiffness. The mean maximum strength was 1,347 ± 357 N for Group A and 1,805 ± 123 N for Group B (p ? 0.0069). The mean deflection was5.54 ± 2.63 mm for Group A and 10.03 ± 3.99 mm for Group B (p ? 0.0056). For the energy variable, the force was 6,203 ± 3,488 N/mm for Group A and 12,885 ± 5,056 N/mm for Group B (p ? 0.0054). Stem B had greater maximum strength, deflection, and energy.

**Conclusions:**

The new stem was effective in neutralizing the impact of axial flexion-compression stresses during biomechanical tests in cadaveric models.

## Introduction

Complication rates after canine total hip replacement (THR) range from 8 to 22% for both cemented and cementless techniques^1^,^2^. Recently, increasing numbers of cases require revision procedures^3^. The most common complications after THR in dogs are infection, aseptic loosening of implants, luxation, femoral fractures, and femoral stem subsidence^4^. Cementless systems are widely used in human patients and have been used in veterinary patients for the past two decades^4^,^5^. One of the reasons for their development was to eliminate the complications associated with the cement^6^. However, in spite of the good performance of canine cementless THR, many studies have described specific complications, especially those associated with the fixation of the femoral component such as catastrophic subsidence, peri-prosthetic fissures and femoral fractures^1^,^2^,^7^.

The longevity of cementless THR depends mainly on effective biological fixation. Anatomical fitting and correct insertion of the femoral stem are critical for bone ingrowth to occur^8^. Two basic types of cementless femoral stems are currently being used in dogs, press-fit and interlock system^4^,^9^,^10^. They have proved to be effective in allowing bone ingrowth^11^,^12^, but subsidence and femoral fractures are still reported^10^,^12^.

A number of femoral stem designs have been described and investigated for use in human THR. They promote better bone ingrowth and, thus, reduce complications. However, there is little information on the different canine femoral THR stem types available^13^. In an attempt to reduce the chance of subsidence or rotation of the femoral stem, a restricting medial collar has been used^1^,^11^. Recently, BioMedtrix has customized the BFX femoral component, including a lateral bolt, to provide additional resistance against subsidence^11^. In order to improve the problems that the stems have been suffering for some decades, new models have been tested to improve the resistance of the implant, improving the mechanical characteristics and the clinical result. Stems with lateral enlargement of the proximal portion were tested, and the results obtained show that these models enabled better filling of the medullary canal, greater resistance to axial forces and greater stability^14^-^17^. As these new designs of veterinary femoral stem are released, aiming to improve anatomical fit and bone ingrowth with reduced complication rates, they require further investigation to determine clinical effectiveness.

The objective of this study was to evaluate the biomechanical properties of a novel total hip replacement femoral stem (Stem B), which was compared to a well-known collared femoral stem (Stem A). The new stem model (Stem B) has as main features a convex proximal portion and a titanium plasma coating. Our hypothesis was that the novel femoral stem would provide more resistance to subsidence and better mechanical performance when compared to the collared stem.

## Methods

### Overall study design and specimen collection

The methodology adopted in this study was approved by the Ethics Committee on Animal Experimentation of Universidade Estadual Paulista “Júlio de Mesquita Filho” (UNESP), under the protocol no. 16,938/16.

**Figure 1:**
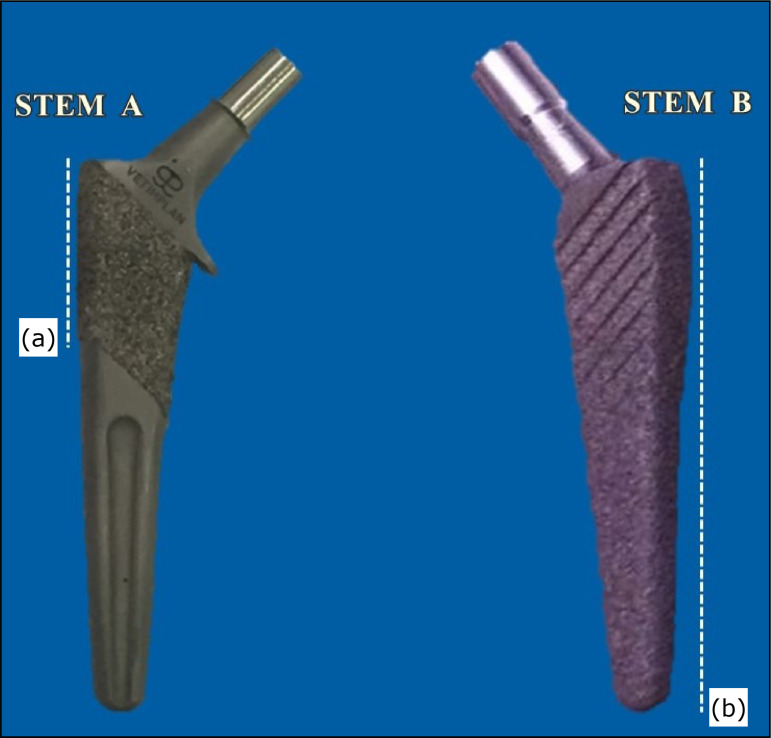
Design of Stem A and Stem B. The dashed lines **(a** and **b)** show that the Stem A has a titanium coating only on its proximal portion, while Stem B is fully coated. Additionally, there is a collar in Stem A. Stem B has a convexity with oblique chamfers on its proximal portion.

The femoral stems (A and B) used in this study were made of surgical stainless steel and coated with a Cr-Co-Mo alloy (ASTM F75) (Cao Medica, Campinas, SP, Brazil). Femoral Stem A has a medial proximal collar and is covered with a titanium plasma coating on its proximal portion. Femoral Stem B has a more domed proximal region and is fully covered with a titanium plasma coating (Fig. 1). Both stems are available in two lengths (Stem A = 65.9 and 71.63 mm and Stem B = 58.54 and 57.34 mm). For this study, a 17-mm-diameter femoral head (neck +0) was used for both stems.

Sixteen femurs from skeletally mature male dog cadavers (25 ± 1 kg), euthanized for reasons unrelated to this study, were used. A randomized comparative experimental design was used, placing implant A in one of the femurs and implant B in the contralateral femur. Each femur was removed from its adjacent soft tissues and wrapped with surgical gauze soaked in a 0.9% NaCl solution. They were then placed in sealed bags and stored at -20ºC.

Radiographic projections (M1) (100 mA, 55 kV, 4 mAs) were taken with a calibration marker of 100 mm. A specific acetate template was used on digital X-rays to determine the size of stem to be implanted in each femur. All samples were subjected to densitometric analysis to ensure uniform bone mineral composition (BMC) and bone mineral density (BMD). The values of BMC and BMD were measured in the X-ray absorptiometry device in two dual energy X-ray absorptiometry (DXA) hologic energies (DXA, Hologic, Marlborough, Massachusetts, United States). This device emits photons that are collimated in a beam that cross the analyzed structure until reaching the detector, in which the intensity of the transferred beam is registered. The mechanism moves from side to side, forming the scan lines that will compose the image. Measurements were made by emitting X-ray beams at different energy levels, creating photoelectric peaks between 80 and 140 kV and 3 mA/s.

In order to check the quality control of the equipment, before scanning, the device was calibrated using a model (hologic DXA quality control spine phantom, hologic, Marlborough, Massachusetts, United States) provided by the manufacturer. This object has an area of 54.4 cm^2^, 51.1 g of BMC and 0.94 g/cm2 of BMD. The specimen was placed on the table, proximal to the flow, and a scan was performed. The data values were automatically computed, and the value obtained, compared with the expected value (previously determined by the manufacturer, at the time of installation). For small samples, less than 50 cm, according to the manufacturer, a second calibration using another model (Rat Step Phanton) is required. Smaller and in a density scale, the object is used to determine the scan area of the sample. If the obtained value differs by more than 1.5% from the expected value, the measurement must be repeated, and maintaining the difference, it is recommended to discontinue the use. The images were analyzed with the aid of the Hologic Discovery Wi10 software (Hologic, Marlborough, Massachusetts, United States). Once the regions of interest are obtained, in this case, the entire sample body of the femur (area) is selected.

It should be noted that all samples were previously weighed and measured, with respect to the length and area of the femur. Weighing was performed using a digital scale and, to measure the length of the femur, a 12-inch digital caliper was used, both properly calibrated. Then, the captured measurements were inserted in the software developed by the equipment manufacturer.

### Stem implantation and instrumentation

The (left and right) femurs were thawed at room temperature. A femoral head and neck ostectomy was made ± 0.5 cm proximal to the lesser trochanter, using a cutting guide and oscillating saw. Using a specific set of rasps and drills, the medullary canal was prepared following a previously described technique^11^. The femoral component was inserted by holding the neck of the implant, without touching the porous parts. The stem was inserted up to 3/4 of the total length of the stem and filled 85 to 90% of the medullary canal. Finally, a specialized impactor was used to push the stem down into the femur to achieve a press-fit fixation.

All specimens were radiographed (M2) before the biomechanical evaluation, to verify correct positioning of the implants.

### Specimen preparation and data processing

**Figure 2:**
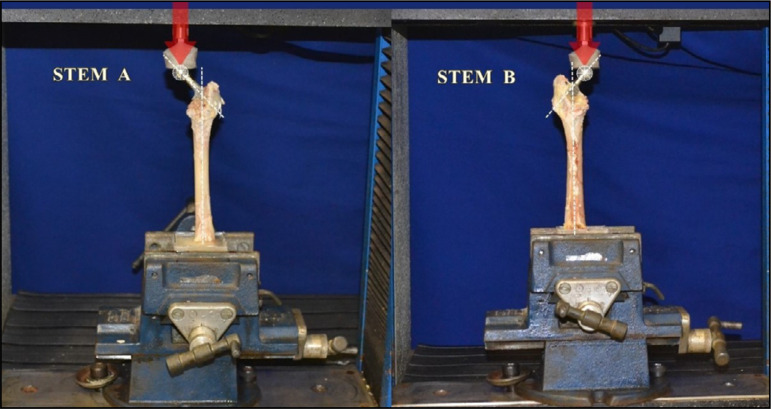
The static flexion-compression test, in which a force of 250 N (red arrow) was used, corresponding to 50% of the maximum force observed during the pilot test. The load cell used was 1,961.33 N (200 kg) and the speed used to promote displacement was 5 mm/min, in the two sample models (A and B).

A force of 250 N was applied to all specimens in the static flexo-compression test, corresponding to 50% of the maximum stress in the pilot test used to determine the strength limit of the system (failure of the construct). An EMIC universal testing machine (maximum capacity of 10,000 kgf, Instron Brasil Equipamentos Científicos, São Jose dos Pinhais, PR, Brazil) was used to deliver a load cell of 1,961.33 N (200 kg). The rate of load was promoted displacement of the stem by 5 mm/min (Fig. 2).

A polymethyl methacrylate support base attached to the distal end of each femur was created to keep the specimens in place. In addition, the acetabular dome (28 mm) was adapted to maintain the specimen in a vertical position, while the flexo-compression stress was applied in the long axis of the samples. The convex surface of the acetabular implant was filled with polymethyl methacrylate through which a hex screw with fine, central threads on its vertex was inserted. The objective was to create a femoral axis at an angle of 15º in the coronal-vertical plane. All tests were performed with the samples at room temperature, average of 22ºC. After the flexion-compression tests, the machine software calculated the maximum force supported by the femur / implant set, the deflection, the relative stiffness, and the energy absorbed during the test for each sample. Failure in the axial compression test was defined as fracture of the specimen.

### Statistical analysis

A randomized block design was used for the comparison of groups (A and B), with two treatments and 16 repetitions (eight for each treatment). Statistics were performed using R software (R Foundation for Statistical Computing, Vienna, Austria). Initially, the homoscedasticity of variances (Bartlett test) and the normal distribution (Shapiro-Wilk test) were tested. The variables from the bone densitometry analysis were compared between groups with the Student’s *t*-test (the independent samples *t* test) to verify the homogeneity of the sample units. The variables obtained from the static axial compression test were subsequently compared between groups through analysis of variance (one-way ANOVA) in a randomized blocks (animal) design. The significance level was set at 95% (p < 0.05) for all tests, and the data was presented as mean ± standard deviation (SD). When the test revealed a statistically significant difference, the Tukey test was also used.

## Results

The pre-test radiographic examinations revealed homogeneity of the samples. The implants were inserted in accordance with the methodological description. The statistical analyses of the variables for BMD and BMC revealed similarity between the selected samples for both Group A and Group B—the samples were homogeneous, and there was no significant difference between groups (p = 0.05) regarding BMD and BMC.

The following variables were obtained during the biomechanical axial static flexo-compression test in the femurs with stems A and B: maximum strength (MS) supported by the implants, deflection (D), stiffness (S) of the construct, and energy (En) applied until failure. Group B had significantly higher values (p ≤ 0.05) for the variables (MS, D, En) resulting from this study, with the exception of S, which was statistically similar between groups (p = 0.05; Table 1).

**Table 1:** Comparison of biomechanical axial flexo-compression test variables between two cementless implant models, Groups A and B. The corresponding values of the resulting variables are expressed as mean (±) and standard deviation (SD).

Variable Flexo-axial compression	Group A	Group B	P-value
Maximum strength (MS) (N)	1.347 ± 357^a^	1.805 ± 123^b^	0.0069^ab^
Deflection(mm)	5.547 ± 2.639^a^	10.033 ± 3.998^b^	0.0056^ab^
Stiffness(N/mm)	860 ± 160^a^	1.011 ± 305^a^	0.2031^ab^
Energy(N.mm)	6.203 ± 3.488^a^	12.885 ± 5.056^b^	0.0054^ab^

a,b Different letters in the columns indicate significant difference, with p ≤ 0.05.

## Discussion

Canine cementless total hip replacement provides excellent clinical outcomes. However, complications such as subsidence, periprosthetic fractures, stress shielding and infections still occur^18^,^19^. Despite improvements in existing systems over recent years^1^,^2^,^11^,^12^, the development of new implants should reduce the currently reported complication rate^20^,^21^. This study aimed to test and compare the biomechanics of a new model of femoral stem with specific characteristics, designed to promote better mechanical performance and adequate adjustment to the femur. The initial hypothesis was that the new femoral stem model (Stem B) would perform well, especially in relation to greater resistance and stress distribution. In fact, Stem B performed better than Stem A.

Some key features are crucial for biomechanical strength of a stem, including the construction material, dimensions and design^22^. The new femoral stem model is coated with a Cr-Co-Mo alloy. This material is widely used in the manufacture of dental and orthopedic implants, mainly for its properties involving biocompatibility, resistance to fatigue and corrosion^23^,^24^. The novel femoral stem (Stem B) has specific characteristics, such as a conical form, a convex and wider proximal portion and fully blasted surface, that result in greater resistance factors. Stem designs focused on a lateral enlargement of the proximal portion have been described for human use, and these were created to maximize the proximal adjustment and the filling of the medullary canal, providing greater torsional and axial stability^14^-^17^. This design potentially increases the contact of the implant in the metaphyseal region, thus increasing the cross-sectional diameter of the stem, allowing the stem to have a broader base of metaphysical support^25^,^26^. As a result, there is a better distribution of endosteal compressive forces, which can potentially reduce complications such as subsidence and aseptic loosening^25^.

Walker *et al*.^28^ compared lateral extended and conventional straight human stems and concluded that the lateral extension resulted in better distribution of periprosthetic stress, allowing the use of smaller stems, facilitating implantation and causing less bone remodeling of the femur. Similarly, Leali and Fetto^27^ demonstrated that larger lateral proximal diameter stems produce an important effect on load transfer to the bone metaphysis. With these stems, the forces between the implant and the bone are concentrated around the level of the lesser trochanter, whereas in straight stems they are located more distally in the diaphysis. Long-term evaluations of these implants confirmed that the change in the proximal geometry provided greater stability and femoral bone preservation, which provides more resistance to migration of the implant. In addition, good femoral canal filling produces high circumferential tensile stresses, that can result in reduced loss of bone^29^.

Despite these positive features of the novel stem design, the lateral and proximal bulging can potentially create an over stress in the proximal femoral canal. Additionally, an over-reaming of the femur is needed, leading to a higher risk for intraoperative fissures and postoperative femoral fracture^29^.

The novel stem has a slightly conical shape, thus differing from the straight conventional stems^30^. Conical-shaped stems have been widely used in human patients, particularly for surgical revisions in which bone loss has occurred in the proximal femur^31^. These provide greater primary rotational stability through continuous pressure in the diaphyseal portion of the bone and better distribution of axial loads and accommodation of the implants in the femoral bone bed, thus reducing complications^30^,^32^,^33^. Additional advantages such as simpler surgical technique, easier intramedullary positioning, better adjustment to different femoral anatomies and simpler correction of retroversion have also been reported^31^,^32^,^34^,^35^. As previously reported, during the implantation of the new stem model (Stem B), and in results of the mechanical tests, there was good coaptation of the implant to the femur. It is possible that this was one of the factors that resulted in the good biomechanical properties.

The coating of implants with materials such as titanium spray plasma assists in the resistance to axial displacement of the stem, allowing a low index of distal micromovement^36^-^38^. Both implants had surface treatment with blasting of glass microspheres and a titanium plasma coating. This coating is thought to provide greater rigidity to the stems (A and B), promoting greater surface contact between the implant and the endosteum. Although the coatings were similar, a higher MS, D and En were observed for construct B. One potential explanation for this is that the differences between groups was due to the extent of the covered area, since Stem B had the coating along its entire length, while the coating of Stem A was restricted to the proximal portion. However, the differences between the proximal regions of each stem, represented by the presence of the collar in Group A, and by the bulging in Group B, might also have resulted in the statistical differences observed.

This research has a number of limitations. More targeted studies are needed to determine whether blasting of glass and titanium plasma microspheres are important for the rigidity and strength of the systems, and whether the extent of the coating is also relevant. Stems A and B in this study were slightly different in size, and it would be interesting to compare the results with a further study using identically sized stems. Additionally, this study was conducted in canine cadaveric models. Therefore, it was not possible to evaluate and compare the effect of the biological fixation obtained by bone growth after the insertion of the stems; further studies in living patients are necessary.

The number of different femoral stem models and designs has been gradually increasing as veterinary medicine advances. However, so far there is no reliable information on the effect of stem design on resistance to subsidence or other complications of total hip prosthesis surgery.

Since the collared femoral stem improves stability and promotes better transfer of stress to the calcar bone^39^, we believe that Stem B can, potentially, behave in the same manner as the collared stem. It is possible that femoral Stem B, due to its larger proximal portion (bulging), would adapt better to the anatomy of the proximal femur, preventing femoral displacement and contributing to adequate filling and osteointegration after implantation^1^,^40^,^41^.

## Conclusions

The new stem model had some results similar to the collar model, mainly by effectively neutralizing the impact of axial flexion-compression stresses on dog femurs. Although the new stem model has achieved superior results to the collar model, and this hypothetically suggests greater mechanical strength, further studies are needed to determine whether the new model is superior mechanically or at least as effective as the model already known.
